# A Functional Genomics View of Gibberellin Metabolism in the Cnidarian Symbiont *Breviolum minutum*

**DOI:** 10.3389/fpls.2022.927200

**Published:** 2022-09-12

**Authors:** Dan Wu, Lin Yang, Jiahua Gu, Danuse Tarkowska, Xiangzi Deng, Qinhua Gan, Wenxu Zhou, Miroslav Strnad, Yandu Lu

**Affiliations:** ^1^State Key Laboratory of Marine Resource Utilization in South China Sea, College of Oceanology, Hainan University, Haikou, China; ^2^Laboratory of Growth Regulators, Palacký University and Institute of Experimental Botany Czech Academy of Sciences, Olomouc, Czechia

**Keywords:** corals, dinoflagellates, environmental stress, gibberellin, zooxanthellae

## Abstract

Dinoflagellate inhabitants of the reef-building corals exchange nutrients and signals with host cells, which often benefit the growth of both partners. Phytohormones serve as central hubs for signal integration between symbiotic microbes and their hosts, allowing appropriate modulation of plant growth and defense in response to various stresses. However, the presence and function of phytohormones in photosynthetic dinoflagellates and their function in the holobionts remain elusive. We hypothesized that endosymbiotic dinoflagellates may produce and employ phytohormones for stress responses. Using the endosymbiont of reef corals *Breviolum minutum* as model, this study aims to exam whether the alga employ analogous signaling systems by an integrated multiomics approach. We show that key gibberellin (GA) biosynthetic genes are widely present in the genomes of the selected dinoflagellate algae. The non-13-hydroxylation pathway is the predominant route for GA biosynthesis and the multifunctional GA dioxygenase in *B. minutum* has distinct substrate preference from high plants. GA biosynthesis is modulated by the investigated bleaching-stimulating stresses at both transcriptional and metabolic levels and the exogenously applied GAs improve the thermal tolerance of the dinoflagellate. Our results demonstrate the innate ability of a selected Symbiodiniaceae to produce the important phytohormone and the active involvement of GAs in the coordination and the integration of the stress response.

## Introduction

Zooxanthellae are a kind of single-celled marine microalgae that live either in the water column as plankton or symbiotically with a variety of marine animals, such as reef-building corals, giant clams, jellyfish, and sea anemones (Jeong et al., 2012). The major symbiotic zooxanthellae are members of the dinoflagellate family Symbiodiniaceae, which has high phylogenetic diversity (Lajeunesse et al., 2018). Annotated genomes are available for several taxa of the family: *Symbiodinium microadriaticum* (Clade A) (Aranda et al., 2016), *Breviolum minutum* (Clade B, formerly *S. minutum*) (Shoguchi et al., 2013), *Cladocopium goreaui* (Clade C, formerly *S. goreaui*) (Liu et al., 2018), and *Fugacium kawagutii* (Clade F, formerly *S. kawagutii*) (Lin et al., 2015). The dinoflagellate endosymbionts supply their cnidarian hosts with photosynthetic products (Kopp et al., 2015) while benefiting from using CO_2_ and nutrients in the host’s waste products (Davy et al., 2012). Although reef-building corals provide numerous socio-economic and ecological benefits in addition to high biodiversity (*inter alia*, tourist and recreational attractions, shoreline protection and fisheries), the global populations of reef systems are declining, partly due to rapid climatic changes, including global warming, ocean acidification, and ultraviolet radiation (Roth, 2014). The loss of algal symbionts or algal pigments is always assumed as critical steps for coral bleaching (Jiang et al., 2021). Therefore, understanding of the stress response and mechanisms underlying these traits of dinoflagellate endosymbionts is critical to the symbiosis stability and the health of the coral systems.

In flowering plants, growth, development, and stress responses are regulated by phytohormones, such as gibberellins (GAs). These hormones regulate crucial and economically relevant processes such as dormancy, germination, growth, and stress responses (Claeys et al., 2014; Hu et al., 2018). Recent evidence suggests that GAs and the associated regulatory mechanisms emerged in an ancient organism before the emergence of land plants (Hu et al., 2018). Orthologs of the GA receptor GID1 have been identified in the bryophyte *Physcomitrella patens* and the lycophyte *Selaginella moellendorffii*, which represent ‘basal’ land plants closest to the green algae (Rensing et al., 2008). Expression of the lycophyte gene in GA receptor mutants of rice (*Oryza sativa*) compensated for their inactive native receptors but the expression of the bryophyte gene did not (Yasumura et al., 2007), suggesting that there are substantial gaps in our understanding of GA evolution. Moreover, accumulating evidence suggests an active involvement of GAs in the biological processes of microalgae (Lu et al., 2010; Piotrowska-Niczyporuk et al., 2012; Stirk et al., 2014), however, it is not known whether the phytohormone is present in symbiotic zooxanthellae, or what functions they may fulfill in those species. Therefore, in this study, we performed a functional genomic study of GA biosynthesis in selected zooxanthellae. Our results show that the GA biosynthesis and inactivation pathways have arisen in dinoflagellate endosymbionts and the GA synthesis is involved in the response of the dinoflagellate *B. minutum* to bleaching-inducing stresses. Our findings improve the understanding of the GA biosynthesis in Symbiodiniaceae, suggest active involvements of GAs during the responses to environmental stimuli, and provide a potential way to alleviate the severity of the mass bleaching of coral reef ecosystems.

## Results and Discussion

### Gibberellin Biosynthesis Is Conserved in the Dinoflagellate Family Symbiodiniaceae

**FIGURE 1:**
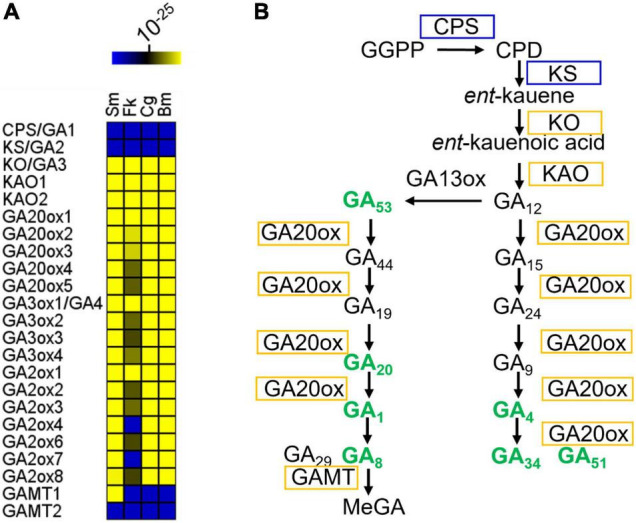
GA biosynthetic pathway in representative Symbiodiniaceae. **(A)** Conservation of GA biosynthetic genes in representative Symbiodiniaceae. The color key indicates the similarity of the gene to its closest match, ranging from low similarity (blue) to high similarity (yellow). Deep blue areas indicate that there were no Blastp hits below the *E*-value threshold (1e-10). For *Arabidopsis* genes with more than one isoform, all homologs with Blastp E-values below 1e-10 were selected for further analysis. If only one gene homologous to any of the *Arabidopsis* isoforms was found in a microalgal genome, it was counted only once. Several algal genes are possibly multi-functional due to their homology to multiple enzymes (Supplementary Dataset 1). **(B)** GA metabolites and biosynthetic pathway in *B. minutum*. Pathways are indicated by black arrows. Enzymes are labeled in boxes. Enzymes labeled in yellow have orthologs in *B. minutum*. Bold green text indicates compounds that are present in *B. minutum*. See Supplementary Table 1 for details. Sm, *Symbiodinium microadriaticum*; Bm, *Breviolum minutum*; Cg, *Cladocopium goreaui*; Fk, *Fugacium kawaguti*; CPS, Ent-copalyl diphosphate synthase; KS, Ent-kaur-16-ene synthase; KO, Ent-kaurene oxidase; KAO1, ent-kaurenoic acid oxidase 1; KAO2, ent-kaurenoic acid oxidase 2; GA20ox1, gibberellin 20 oxidase 1; GA3ox1, gibberellin 3-beta-dioxygenase 1; GAMT, S-adenosyl-L-methionine-dependent methyltransferases-like protein; GGPP, geranylgeranylpyrophosphate; CPD, ent-copalyl diphosphate; MeGA, methylated GAs.

The GA biosynthetic pathway can be divided into three parts. In the first part, geranylgeranylpyrophosphate (GGPP) is converted into *ent*-copalyl diphosphate by *ent*-copalyl diphosphate synthase (CPS or GA1) (Sun and Kamiya, 1994) and then into *ent*-kaurene by *ent*-kaurene synthase (KS or GA2) (Yamaguchi et al., 1998; Figure 1). Neither *Arabidopsis* GA1 nor GA2 orthologs were identified in the sampled dinoflagellates, including *Symbiodinium microadriaticum*, *Breviolum minutum*, *Cladocopium goreaui*, and *Fugacium kawaguti* (Figure 1A). Unlike vascular plants, fungi catalyze *ent*-kaurene formation from GGPP using a single bifunctional CPS/KS enzyme (Tudzynski et al., 1998). Such a bifunctional enzyme has not been identified in these algae. However, genes encoding *ent*-kaurene oxidase (KO or GA3) and *ent*-kaurenoic acid oxidase (KAO) (Helliwell et al., 1999), the enzymes from the second part of the GA-biosynthetic pathway, were identified in every species examined here (Figure 1A). In the third part of the pathway, GA_12_ and GA_53_ are further oxidized to other C_20_- and C_19_-GAs by a series of 2-oxoglutarate-dependent dioxygenases (2ODDs) (Lange et al., 1994; Israelsson et al., 2004; Figure 1B). In *Arabidopsis*, the 2ODDs in each of the three families (17 genes in total) act as biosynthetic and catabolic enzymes, respectively. However, far fewer putative 2ODD genes were found in the dinoflagellates. Thus the GA biosynthetic pathway is moderately conserved in evolutionary terms and occur in dinoflagellate species.

### Symbiodiniaceae Has Different Dominant Gibberellin Species and Biosynthetic Intermediates Compared With Those of Higher Plants

To validate the genome-based metabolic reconstruction, we then selected *B. minutum* as a model to exam the presence of GA metabolites in the dinoflagellates. This species is an emerging research model for study on the mutualistic partnerships between dinoflagellate endosymbionts and cnidarian hosts (Davy et al., 2012; Lu et al., 2020; Zhou et al., 2022). Ultra-performance liquid chromatography-electrospray ionization tandem mass spectrometry (UPLC-ESI-MS/MS) revealed that *B. minutum* contained significant quantities of GAs, with GA51 being the most abundant (Figure 1B and Supplementary Table 1). It thus seems that the GA-synthesis pathway in microalgae involves as-yet-unidentified CPSs and KSs (or may involve an entirely novel route). This is supported by the finding that *Arabidopsis ga1* null mutants (GA1 is the single-copy gene that encodes *ent*-copalyl diphosphate synthase; *i.e.*, CPS), although very severely dwarfed, contain trace quantities of various GAs (Sponsel and Hedden, 2010). GA_4_ and GA_1_ are both present in *B. minutum* (Figure 1B and Supplementary Table 1) and are known to be bioactive in many species including *Arabidopsis*. GA_4_ and GA_1_ are formed from GA_9_ and GA_20_, respectively, in plants by the action of GA3 hydroxylase (GA3ox). GA_12_ and GA_53_ are the precursors of GA_9_ and GA_20_, respectively, and are formed by the action of GA20ox (Figure 1B). In *B. minutum*, GA_53_ and GA_20_ were identified whereas GA_12_ and GA_9_ were absent (Figure 1B and Supplementary Table 1).

In many plants, GA_12_ is converted to GA_53_ by a GA13ox-catalyzed hydroxylation at C-13. In most plants, the 13-hydroxylation pathway predominates, although in *Arabidopsis* and some other species, the non-13-OH-pathway is more prevalent (Zanewich and Rood, 1995). In the cytosol, GA_53_ is converted into various other GAs via parallel pathways. This conversion involves a series of oxidations at C-20, resulting in the eventual loss of C-20 and the formation of C_19_-GAs. In the non-13-hydroxylation pathway, GA_12_ is oxidized to produce GA_9_ (both are absent in *B. minutum*), via the intermediacy of GA_15_ and GA_24_ (both are absent) (Figure 1B and Supplementary Table 1). GA_9_ is then oxidized to the bioactive compound GA_4_ (present) by a 3β-hydroxylation reaction (Figure 1B and Supplementary Table 1). In the 13-hydroxylation pathway, GA_53_ is sequentially oxidized at C-20 to produce GA_20_ (present), which is then 3β-hydroxylated to yield bioactive GA_1_ (present) (Figure 1B and Supplementary Table 1). Finally, hydroxylation at C-2 converts GA_4_ and GA_1_ into their inactive forms, GA_34_ (present), GA_51_ (present), GA_29_ (absent) and GA_8_ (present), respectively (Figure 1B and Supplementary Table 1). Therefore, most intermediates from the 13-OH pathway for the synthesis of bioactive GA_1_ are present in *B. minutum*, along with intermediates from their deactivation pathways. In contrast, the intermediates from the non-13-OH pathway are absent, but bioactive GA_4_ and its deactivation forms (GA_34_ and GA_51_) are present (Figure 1B). Moreover, the amount of GA_51_ is relatively higher than the remaining GA forms (Supplementary Table 1). These results may suggest that the non-13-hydroxylation pathway may predominate in *B. minutum* and the multifunctional GA dioxygenase in *B. minutum* has a distinct substrate preference from those in *Arabidopsis*.

### Gibberellin Synthesis Is Involved in Various Stress Responses in *Breviolum minutum*

**FIGURE 2:**
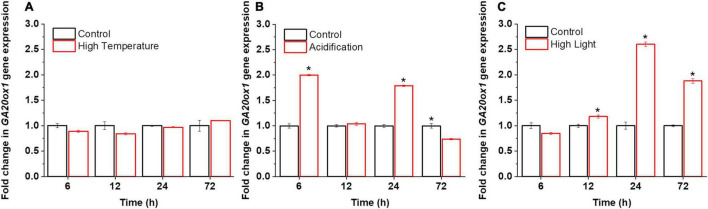
Transcriptional dynamics of GA biosynthetic gene *GA_20_ox1* in *B. minutum* during the stress responses. **(A)** Transcriptional dynamics of *GA_20_ox1* in *B. minutum* under the thermal stress. **(B)** Transcriptional dynamics of *GA_20_ox1* in *B. minutum* under the acidification stress. **(C)** Transcriptional dynamics of *GA_20_ox1* in *B. minutum* under the high light stress. Data are presented as means ± SDs (*n* = 4). The asterisks (*) indicate statistically significant differences (*P* ≤ 0.05).

Little was known about the functional roles of GAs in microalgae, particularly at the molecular level. Although the application of exogenous GAs to microalgal cultures suggests that they may function as stress signals (Park et al., 2013; González-Garcinuño et al., 2016; Yu et al., 2016), the evidence is still inconclusive. We thus probed the possible effects of abiotic stresses on the synthesis of GAs in dinoflagellate endosymbionts. For reef-building corals, the bleaching process is induced by environmental stimuli, such as high light, high temperature, and acidification (Lu et al., 2020; Jiang et al., 2021). Therefore, to probe the physiological relevance of GAs in microalgae, we monitored the transcriptional activities of the committed GA biosynthetic gene GA 20-oxidase (*GA_20_ox1*) and GA profiles under the high light, high temperature, and acidification conditions. These time-series datasets unveiled the active involvement and the dynamics of GAs and GA-related transcripts during stress responses in *B. minutum* (Figure 2).

In higher plants, GAs play direct or indirect roles in plant responses to abiotic stress (Peleg and Blumwald, 2011). In *B. minutum*, under the thermal stress, the transcription of *GA_20_ox1* was largely stable where it declined somewhat after the stress onset (*p* > 0.05; 6 h) and then return to the control level (24 h) (Figure 2A). In contrast, either high light or acidification increased the abundance of *GA_20_ox1* transcripts somehow (Figures 2B,C). Upregulation of *GA_20_ox1* occurred rapidly within the first 6 h upon acidification, with its transcript levels at a relatively higher level at 24 h (Figure 2B). Nevertheless, a depressed level of *GA_20_ox1* transcript was observed following long-term acidification (72 h; Figure 2B). Following high light, the transcript levels of *GA_20_ox1* follow a trend that declined at the early stage and increased slowly and continuously afterward, peaking at 24 h (Figure 2C). This suggested that, upon high light and acidification, relative to high temperature, the *GA_20_ox1* gene may contribute more to stress response at the transcriptional levels. The rate of GA biosynthesis is thus regulated as part of the response to the bleaching-inducing environmental stimuli. This strongly suggests that GA-based processes are important for at least the explored stress conditions in *B. minutum*, which is consistent with the alleviation stress symptoms of green microalga *Chlorella vulgaris* by exogenously applied GA_3_ (Piotrowskaniczyporuk et al., 2012).

**FIGURE 3:**
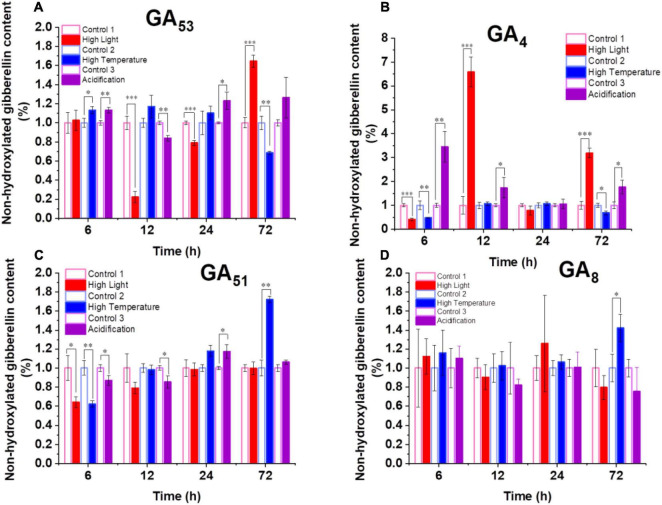
Dynamics of GAs in *B. minutum* during the high-light, high-temperature, and acidification stresses. **(A)** Dynamics of GA_53_, **(B)** Dynamics of GA_4_, **(C)** Dynamics of GA_51_, **(D)** Dynamics of GA_8_. The asterisks (*), (^**^), and (^***^) indicate statistically significant differences with *P* ≤ 0.05, *P* ≤ 0.01, and *P* ≤ 0.001, respectively.

The increased transcription of GA biosynthesis genes in *B. minutum* does not always equal to increased biosynthesis of GAs. We thus probed the dynamics of intracellular GA levels under the three selected stresses. To assess the dynamic equilibrium of 13-OH and non-13-OH pathways for GA biosynthesis, we further determined the detailed GA profiling. Despite of the fluctuation, with long-term stress treatments (72 h), levels of GA_53_ increased under the high-light and acidification conditions while it decreased under high temperature (Figure 3A). The levels of the known bioactive species GA_1_ generated through the 13-OH pathway were not stable (could be detected at limited time points). In contrast, although the accumulation of GA_12_ could not be detected under either control or stressed conditions, the bioactive product of the non-13-OH pathway (*i.e.*, GA_4_) was remarkably decreased following the onset of high light treatment (P ≤ 0.001) and returned to a level higher than the control after 72 h treatment (Figure 3B). As for the acidification conditions, the level of GA_4_ was rapidly elevated within the first 6 h and remained at a relatively higher level than the control thereafter (Figure 3B). This was consistent with the transcriptional abundance of *GA_20_ox1* under the high light and acidification conditions where the *GA_20_ox1* transcript increased at the early stage upon acidification while it declined at the early stage and increased under prolonged high light conditions (Figure 2B,C). For the inactive GA forms in the non-13-OH pathways, GA_51_ was rapidly decreased following the onset of all three stresses (Figure 3C) while the level of GA_34_ was relatively similar between the stressed algal cells and the controls (Supplementary Table 1). Meanwhile, inactive forms in the 13-OH pathways (*i.e.*, GA_8_) remained at relative stable levels under high-light and acidification treatments (Figure 3D). In contrast, under thermal stress, the content of GA_8_ increased at the end of detection (Figure 3D). Therefore, both the biosynthesis and deactivation pathways are actively involved in the stress responses and the non-13-hydroxylation pathway played predominant roles in GA homeostasis in *B. minutum*. Moreover, despite fluctuations and wide discrepancy in the levels of various GA species, the content of the active GA species (*i.e.*, GA_4_ at late phase) increased upon the high-light and acidification stresses (Figure 3B). In contrast, GA_4_ decreased at the end of detection under the thermal stress (Figure 3B). These observations suggest a general function and a stress-dependent manner of GAs in the dinoflagellate endosymbiont.

### Exogenously Applied Gibberellins Improves the Thermal Tolerance of *Breviolum minutum*

**FIGURE 4:**
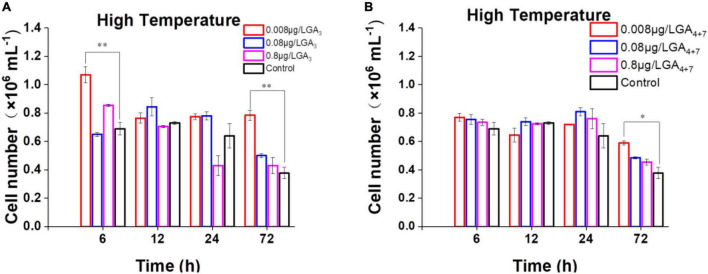
Effects of GAs on the growth of *B. minutum* cells under the thermal stress. **(A)** Effects of GA_3_ on the growth of *B. minutum* cells. **(B)** Effects of GA_4+7_ on the growth of *B. minutum* cells. GA_4+7_, a mixture of GA_4_ and GA_7_. The asterisks (*) and (^**^) indicate statistically significant differences with *P* ≤ 0.05 and *P* ≤ 0.01, respectively.

More than 130 GAs have been identified in plants while only a handful of them are biologically active in flowering plants which include GA_1_, GA_3_, GA_7_, and GA_4_ (∼1000 fold higher activity than GA_1_) (Kapoore et al., 2021). Among the active forms, GA_3_ and GA_4+7_ (a mixture of GA_4_ and GA_7_) are commercially available, we thus select them to further probe the potential functions of GAs in *B. minutum*. The levels of GAs were perturbed by supplementing GA_3_ and GA_4+7_ to algal cultures at concentrations of 0.008, 0.08, and 0.8 μg⋅L^–1^. For GA-treated and untreated cultures, algal cells at the linear growth phase were inoculated into the fresh medium under the indicated conditions, and the cell growth was tracked for three days. Although subtle difference was observed between the algal cells treated with or without GAs under the control conditions (pH 8.2, 25°C, and 50 μmol⋅photons⋅m^–2^⋅s^–1^ light intensity), the growth was higher than the control under the thermal stress in either GA_3_- (Figure 4A) or GA_4+7_- (Figure 4B) treated cells (*p* ≤ 0.05). Exogenously applied GAs may compensate for the decreased endogenous GAs under the thermal stress (*i.e.*, GA_4_; Figure 3B) which subsequently improved the dinoflagellate’s growth. To assess whether GAs plays a role in the response to environmental stresses other than high temperature, the algal cells in the linear growth phase were inoculated into the fresh medium with the presence or absence of GAs under the high-light or the acidification conditions. However, a bare difference was observed in cell numbers between the GA-treated and the untreated cells till the end of detection. This might attribute to that the amount of GA_4_ under these stresses is already higher than that of the controls (Figure 3B). Therefore, GAs could act as a stress hormone in *B. minutum* where their effects are stress- and time-dependent.

## Conclusion

In summary, the endosymbiotic dinoflagellates produce a wide range of GAs using relatively conserved biosynthetic enzymes. In the endosymbiont of reef corals *B. minutum*, these hormones are involved in the response of the bleaching-inducible environmental stresses, specifically, the exogenous applied GAs could improve the thermal tolerance of the dinoflagellate. Dissection of the mechanism related might facilitate the improvement of stress tolerance of endosymbiotic dinoflagellates and provide a way for the control and the remedy of massive coral bleaching. However, to fully exploit this technique potential in ecosystem restoration, many questions remain to be answered. First, how to apply the hormone technique in the highly variable open ocean (Jiang et al., 2021). Second, a genetic manipulation of crucial GA-relating genes (e.g., GA20ox) of the dinoflagellates by genome editing technology would further improve the practice (Jiang et al., 2021; Lu et al., 2021b).

## Materials and Methods

### Culture Conditions and Physiological Measurements

*Breviolum minutum* was purchased from the National Institute for Environmental Studies (Japan). They were cultured routinely in 250-ml conical flasks with L1 medium containing kanamycin (50 mg⋅L^–1^), and streptomycin (50 mg⋅L^–1^) to minimize the growth of bacteria (Lu et al., 2020). When culturing dinoflagellate cells, the control group was maintained under the organism’s preferred physiological conditions (pH 8.2, 25°C, and continuous 50 μmol⋅photons⋅m^–2^⋅s^–1^ light intensity) as described in earlier reports (Jiang and Lu, 2019). Under otherwise identical high-light, high-temperature and acidification stress conditions, the light intensity, temperature, and pH were set at 200 μmol⋅photons⋅m^–2^⋅s^–1^, 34°C, and pH 7.6, respectively, based on our previous tests (Jiang and Lu, 2019; Lu et al., 2020). Algal cells at a concentration of approximately 1 × 10^6^ cells⋅ml^–1^ were harvested, washed with sterile seawater, and inoculated into the fresh medium in triplicate. Cultures were started with the same initial cell concentration of 2 × 10^5^ cells⋅ml^–1^, acclimated for 12 h under 50 μmol⋅photons⋅m^–2^⋅s^–1^ light, and then exposed to designated stress conditions. Growth was monitored by measuring the turbidity (Biochrom GeneQuant 1300, GE Healthcare) and the cell number (LUNA-FL Dual Fluorescence Cell Counter, Logos Biosystems) at indicated intervals (Gan et al., 2017; Lu et al., 2020).

### Gene Expression Analysis

The cDNA preparation and reverse transcription were performed as previously (Lu et al., 2021a). In brief, the threshold cycle (2-^–ΔΔ*Ct*^) method was used to quantify relative changes in transcript levels from the quantitative PCR (qPCR) data. Levels of the transcripts under each set of treatment conditions at each time point were first normalized to actin expression levels. The values obtained for each gene were then normalized to the values in the control treatments (specimens that were not subjected to the stresses) at the corresponding time point. Values are means and standard errors obtained from three experiments (Han et al., 2020). The primers used are listed in Supplementary Table 2.

### Gibberellins Analysis

The sample preparation and analysis of GAs was performed according to the previous report (Urbanová et al., 2013) with some modifications. Briefly, the lyophilized algal materials (100 mg dry weight) were sonicated and extracted with 1 mL 80% acetonitrile containing 5% formic acid and 19 internal gibberellin standards ([^2^H_2_]GA_1_, [^2^H_2_]GA_3_, [^2^H_2_]GA_4_, [^2^H_2_]GA_5_, [^2^H_2_]GA_6_, [^2^H_2_]GA_7_, [^2^H_2_]GA_8_, [^2^H_2_]GA_9_, [^2^H_2_]GA_12_, [^2^H_2_]GA_12_ald, [^2^H_2_]GA_15_, [^2^H_2_]GA_19_, [^2^H_2_]GA_20_, [^2^H_2_]GA_24_, [^2^H_2_]GA_29_, [^2^H_2_]GA_34_, [^2^H_2_]GA_44_, [^2^H_2_]GA_51_, [^2^H_2_]GA_53_). The supernatant was collected and purified using mixed mode reversed-phase and ion-exchange cartridges (Waters, Milford, MA, United States) and analyzed by UPLC-MS/MS (Micromass, Manchester, United Kingdom). GAs were detected using a multiple-reaction monitoring mode of the transition of the ion [M–H]^–^ to the appropriate product ion. Masslynx 4.2 software (Waters, Milford, MA, United States) was used to analyze the data.

### Exogenous Application of Gibberellins

Exogenous application experiments were conducted according to the early report (Lu et al., 2014). Cells were synchronized by alternating light/dark (12/12 h) cycles with a final extended dark period. Synchronous cells were added with GA_3_ or GA_4+7_ at a gradient concentration (0.008, 0.08, 0.8, 8 μg⋅L^–1^) or an equivalent amount of ethanol (not to exceed a final concentration of 0.1%). Samples were taken at the indicated periods for further analysis.

### Data Sources and Statistical Analyses

Genome sequences for dinoflagellates were retrieved from http://reefgenomics.org. Proteins from all genomes were blasted with the validated *Arabidopsis* proteins. The sequences that were ultimately selected are listed in Supplementary Dataset 1. All the experiments were carried out in triplicate, statistical analyses were done using SPSS 18.0 software, standard deviation analysis was used to compare mean values of replicate data sets. Significant difference analysis was undertaken by using Tukey’s test in Graph Prism Two-Way ANOVA.

## Data Availability Statement

The original contributions presented in this study are included in the article/Supplementary Material, further inquiries can be directed to the corresponding author.

## Author Contributions

YL contributed to the designing experiments. DW contributed to the performing experiments related to exogenous application of GAs. DT contributed to the performing experiments related to the GA measurements. QG contributed to the performing experiments related to gene expression. LY, JG, and XD contributed to the sample collections. YL, WZ, MS, and DT wrote the manuscript. All authors contributed to the article and approved the submitted version.

## Conflict of Interest

The authors declare that the research was conducted in the absence of any commercial or financial relationships that could be construed as a potential conflict of interest.

## Publisher’s Note

All claims expressed in this article are solely those of the authors and do not necessarily represent those of their affiliated organizations, or those of the publisher, the editors and the reviewers. Any product that may be evaluated in this article, or claim that may be made by its manufacturer, is not guaranteed or endorsed by the publisher.
