# Supplementary material for: A Functional Genomics View of Gibberellin Metabolism in the Cnidarian Symbiont Breviolum minutum
Source: Front Plant Sci. 2022 Sep 12;13:927200. doi: 10.3389/fpls.2022.927200 (PMC9510744; doi:10.3389/fpls.2022.927200)
Supplement: Supplementary file 2 [file Table_1.docx]

**Table S1**. Quantification of gibberellins (GAs) in *B. minutum*.

| **GAs (pg/mgDW)** | | **High light** | | **High temperature** | | **Acidification** | |
| --- | --- | --- | --- | --- | --- | --- | --- |
|  |  | **Control** | **Treatment** | **Control** | **Treatment** | **Control** | **Treatment** |
| **GA_1_** | 6h | not detected | not detected | not detected | not detected | not detected | not detected |
|  | 12h | not detected | not detected | 2.77±1.10 | 1.54±0.78 | not detected | not detected |
|  | 24h | not detected | not detected | 1.39±0.28 | not detected | 0.38±0.08 | not detected |
|  | 72h | not detected | not detected | 5.88±1.13 | not detected | not detected | not detected |
| **GA_3_** | 6h | 1.38±0.28 | 0.78±0.27 | 1.26±0.09 | 0.98±0.14 | 1.30±0.34 | 1.19±0.29 |
|  | 12h | 1.43±0.31 | 1.44±0.37 | 1.45±0.27 | 1.46±0.06 | 0.82±0.21 | 0.93±0.16 |
|  | 24h | 1.39±0.19 | 1.30±0.57 | 1.62±0.70 | 1.16±0.27 | 1.12±0.18 | 1.17±0.14 |
|  | 72h | 0.95±0.24 | 1.08±0.10 | 0.98±0.37 | 1.55±0.11 | 1.01±0.08 | 0.77±0.14 |
| **GA_4_** | 6h | 3.32±0.09 | 1.35±0.14 | 2.52±0.37 | 1.21±0.05 | 0.73±0.05 | 2.52±0.38 |
|  | 12h | 0.51±0.16 | 3.36±0.26 | 2.48±0.27 | 2.65±0.17 | 0.94±0.05 | 1.63±0.33 |
|  | 24h | 2.98±0.16 | 2.32±0.46 | 3.35±0.30 | 3.57±0.19 | 1.07±0.05 | 1.13±0.18 |
|  | 72h | 0.85±0.11 | 2.71±0.14 | 2.70±0.20 | 1.85±0.16 | 1.42±0.17 | 2.51±0.33 |
| **GA_8_** | 6h | 1.63±0.44 | 1.83±0.25 | 2.13±0.41 | 2.47±0.15 | 2.39±0.40 | 2.63±0.25 |
|  | 12h | 2.41±0.20 | 2.18±0.26 | 2.55±0.31 | 2.61±0.40 | 2.76±0.61 | 2.27±0.14 |
|  | 24h | 1.86±0.20 | 2.34±0.77 | 2.54±0.16 | 2.70±0.15 | 2.47±0.18 | 2.49±0.32 |
|  | 72h | 2.90±0.47 | 2.31±0.30 | 2.42±0.28 | 3.44±0.05 | 3.09±0.23 | 2.33±0.63 |
| **GA_13_** | 6h | 0.23±0.01 | 0.39±0.02 | 0.29±0.07 | 0.20±0.04 | 0.10±0.01 | 0.12±0.03 |
|  | 12h | 0.15±0.04 | 0.42±0.07 | 0.07±0.02 | 0.27±0.01 | 0.23±0.02 | 0.14±0.01 |
|  | 24h | 0.26±0.02 | 0.20±0.04 | 0.45±0.04 | 0.31±0.01 | 0.14±0.01 | 0.22±0.01 |
|  | 72h | 0.32±0.04 | 0.15±0.02 | 0.39±0.01 | 0.24±0.03 | 0.05±0.03 | 0.13±0.03 |
| **GA_19_** | 6h | 1.91±0.09 | 1.62±0.25 | 2.05±0.19 | 1.26±0.11 | 1.46±0.12 | 1.68±0.15 |
|  | 12h | 1.91±0.11 | 2.01±0.13 | 1.6±0.21 | 1.65±0.39 | 1.49±0.22 | 1.26±0.16 |
|  | 24h | 1.80±0.24 | 1.38±0.40 | 1.45±0.07 | 1.68±0.20 | 1.58±0.11 | 1.45±0.15 |
|  | 72h | 1.46±0.20 | 2.15±0.03 | 1.75±0.26 | 1.89±0.09 | 1.22±0.17 | 0.93±0.07 |
| **GA_20_** | 6h | 0.31±0.04 | not detected | 0.72±0.02 | 0.15±0.03 | 0.51±0.07 | 0.39±0.04 |
|  | 12h | 0.60±0.02 | 0.33±0.04 | 0.08±0.01 | 0.09±0.01 | 0.14±0.02 | 0.28±0.01 |
|  | 24h | 0.04±0.00 | 0.72±0.05 | 0.74±0.03 | 1.45±0.27 | 0.05±0.01 | 0.27±0.02 |
|  | 72h | 0.31±0.02 | 0.23±0.04 | 0.97±0.04 | 3.50±0.32 | 0.15±0.01 | 0.34±0.02 |
| **GA_34_** | 6h | 2.73±0.23 | 2.53±0.63 | 2.91±0.12 | 2.78±0.37 | 2.67±0.14 | 2.7±0.21 |
|  | 12h | 2.65±0.24 | 2.53±0.36 | 2.7±0.43 | 2.81±0.21 | 2.32±0.15 | 2.36±0.15 |
|  | 24h | 2.55±0.15 | 2.32±0.21 | 2.65±0.32 | 2.67±0.41 | 2.81±0.12 | 2.62±0.29 |
|  | 72h | 2.77±0.14 | 2.98±0.02 | 2.74±0.34 | 2.98±0.10 | 2.88±0.62 | 2.64±0.32 |
| **GA_44_** | 6h | 1.91±0.29 | 1.24±0.22 | 2.67±0.21 | 2.31±0.34 | 1.43±0.29 | 2.32±0.24 |
|  | 12h | 2.47±0.32 | 3.06±0.43 | 1.72±0.23 | 0.63±0.13 | 3.33±0.39 | 1.85±0.36 |
|  | 24h | 4.12±0.35 | 3.23±0.15 | 2.38±0.28 | 1.40±0.16 | 1.46±0.07 | 1.88±0.46 |
|  | 72h | 2.27±0.21 | 0.81±0.09 | 4.36±0.27 | 2.21±0.11 | 1.54±0.23 | 1.28±0.32 |
| **GA_51_** | 6h | 433.11±57.09 | 278.42±20.76 | 1081.86±66.95 | 672.74±29.33 | 739.25±21.88 | 642.77±32.34 |
|  | 12h | 316.67±38.67 | 250.57±15.38 | 632.56±24.28 | 621.17±25.04 | 538.43±10.48 | 459.46±29.00 |
|  | 24h | 357.45±25.35 | 352.15±19.78 | 608.76±17.32 | 641.38±30.58 | 718.02±10.50 | 842.51±41.41 |
|  | 72h | 337.59±11.02 | 337.07±18.29 | 78.98±5.21 | 13.3±1.86 | 1358.8±37.30 | 1443.8±23.47 |
| **GA_53_** | 6h | 8.63±0.56 | 8.86±0.75 | 7.57±0.29 | 8.57±0.24 | 8.35±0.18 | 9.44±0.19 |
|  | 12h | 8.02±0.47 | 1.79±0.38 | 10.64±0.40 | 12.46±1.03 | 11.31±0.16 | 9.51±0.27 |
|  | 24h | 9.68±0.16 | 7.65±0.21 | 13.06±1.32 | 14.39±0.76 | 8.82±0.07 | 10.85±0.65 |
|  | 72h | 4.51±0.20 | 7.42±0.23 | 14.27±0.81 | 9.78±0.15 | 11.12±0.32 | 14.07±1.92 |
