# Supplementary material for: A Functional Genomics View of Gibberellin Metabolism in the Cnidarian Symbiont Breviolum minutum
Source: Front Plant Sci. 2022 Sep 12;13:927200. doi: 10.3389/fpls.2022.927200 (PMC9510744; doi:10.3389/fpls.2022.927200)
Supplement: Supplementary file 3 [file Table_2.docx]

**Table S2**. Primers used in this study.

| **Primer** | **Oligonucleotide Sequence 5’-3’'** | **Product Size(bp)** |
| --- | --- | --- |
| *Actin*--F | CAACGGAAGTGGAATGTGC | 307 |
| *Actin*-R | CTTTGGGTTCAAGGGTGC |  |
| *GA_20_oxl*-F | TATTCCGCACTACCAGTCCTACCG | 229 |
| GA_20_oxl-R | TGTTGGACATCGCAATGCTCTACT |  |
